# The Contribution of Homocysteine Metabolism Disruption to Endothelial Dysfunction: State-of-the-Art

**DOI:** 10.3390/ijms20040867

**Published:** 2019-02-17

**Authors:** Ruben Esse, Madalena Barroso, Isabel Tavares de Almeida, Rita Castro

**Affiliations:** 1 Department of Biochemistry, Boston University School of Medicine, Boston, MA 02118, USA; ruben.esse@gmail.com; 2 University Children’s Research@Kinder-UKE, University Medical Center Hamburg-Eppendorf, 20246 Hamburg, Germany; m.barroso@uke.de; 3 Laboratory of Metabolism and Genetics, Faculty of Pharmacy, University of Lisbon, 1649-003 Lisbon, Portugal; italmeida@ff.ul.pt; 4 Institute for Medicines and Pharmaceutical Sciences (iMed.UL), Faculty of Pharmacy, University of Lisbon, 1649-003 Lisbon, Portugal; 5 Department of Biochemistry and Human Biology, Faculty of Pharmacy, University of Lisbon, 1649-003 Lisbon, Portugal; 6 Department of Nutritional Sciences, The Pennsylvania State University, University Park, PA 16802, USA

**Keywords:** *S*-adenosylhomocysteine, cellular methylation, atherosclerosis

## Abstract

Homocysteine (Hcy) is a sulfur-containing non-proteinogenic amino acid formed during the metabolism of the essential amino acid methionine. Hcy is considered a risk factor for atherosclerosis and cardiovascular disease (CVD), but the molecular basis of these associations remains elusive. The impairment of endothelial function, a key initial event in the setting of atherosclerosis and CVD, is recurrently observed in hyperhomocysteinemia (HHcy). Various observations may explain the vascular toxicity associated with HHcy. For instance, Hcy interferes with the production of nitric oxide (NO), a gaseous master regulator of endothelial homeostasis. Moreover, Hcy deregulates the signaling pathways associated with another essential endothelial gasotransmitter: hydrogen sulfide. Hcy also mediates the loss of critical endothelial antioxidant systems and increases the intracellular concentration of reactive oxygen species (ROS) yielding oxidative stress. ROS disturb lipoprotein metabolism, contributing to the growth of atherosclerotic vascular lesions. Moreover, excess Hcy maybe be indirectly incorporated into proteins, a process referred to as protein N-homocysteinylation, inducing vascular damage. Lastly, cellular hypomethylation caused by build-up of *S*-adenosylhomocysteine (AdoHcy) also contributes to the molecular basis of Hcy-induced vascular toxicity, a mechanism that has merited our attention in particular. AdoHcy is the metabolic precursor of Hcy, which accumulates in the setting of HHcy and is a negative regulator of most cell methyltransferases. In this review, we examine the biosynthesis and catabolism of Hcy and critically revise recent findings linking disruption of this metabolism and endothelial dysfunction, emphasizing the impact of HHcy on endothelial cell methylation status.

## 1. Introduction

Cardiovascular diseases (CVD) are a prominent cause of mortality [1,2]. The leading cause of CVD is atherosclerosis that is elicited by the impairment of endothelial function, or endothelial dysfunction, and results in a chronic condition in which arteries harden through the build-up of plaques in the vessel wall [3]. Several studies show that elevated homocysteine (Hcy) promotes endothelial dysfunction and atherosclerosis [4,5]. Hcy is derived from the essential amino acid methionine, which serves not only as a building block for protein synthesis, but also as precursor of S-adenosylmethionine (AdoMet) required for almost all transmethylation reactions occurring in a cell. The association between Hcy and CVD risk may be rooted, at least partially, in the defective cellular methyl transfer processes that accompany the intracellular accumulation of Hcy [6]. However, other mechanisms may also explain the vascular toxicity associated with HHcy [7]. Here we present an overview of the latest and most significant evidence about Hcy metabolism disruption and CVD, focusing on the different pathophysiological mechanisms underlying this association.

## 2. The Methionine Metabolism and Cell Methylation Status

Transmethylations are biologically critical chemical reactions in which a methyl group is transferred from one compound to another. In mammals, these reactions are generally regulated by the intracellular concentration of the compound that donates the methyl group, S-adenosylmethionine (AdoMet), and by that of the compound formed after the reaction takes place, S-adenosylhomocysteine (AdoHcy) [8]. AdoHcy inhibits the activity of the majority of AdoMet-dependent methyltransferases, thus AdoMet and AdoHcy concentrations determine a cell’s methylation balance [8,9].

**Figure 1:**
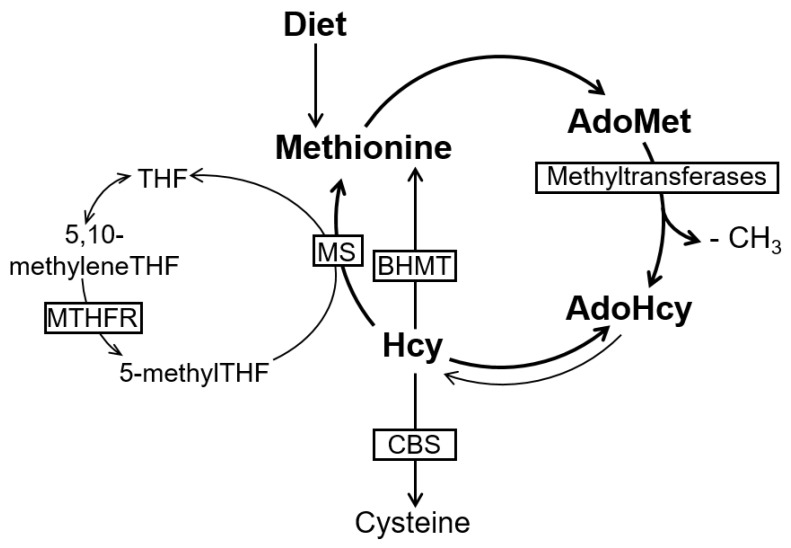
Simplified diagram of methionine metabolism (adapted from [22]). Methionine is converted to S-adenosylmethionine (AdoMet) by ATP-L-methionine S-adenosyltransferase. AdoMet, serves as methyl group donor for methylation of DNA, proteins, and other biomolecules, generating S-adenosylhomocysteine (AdoHcy), which is hydrolyzed to homocysteine (Hcy) and adenosine. This hydrolysis is reversible, and AdoHcy synthesis is favored rather than its hydrolysis. Nevertheless, under normal conditions, Hcy will be rapidly removed ensuring the hydrolytic direction. The biochemical removal of Hcy is either through the transsulfuration pathway, whose rate-limiting step is catalyzed by cystathionine β-synthase (CBS), or by its remethylation to methionine. This remethylation can be folate-dependent (requiring the enzymatic activities of methionine synthase (MS) and 5,10-methylenetetrahydrofolate reductase (MTHFR)) or folate-independent (requiring the enzymatic activity of betaine-homocysteine methyltransferase (BHMT)).

AdoMet and AdoHcy are formed during the metabolism of the essential amino acid methionine (Figure 1) [4]. More specifically, AdoMet is formed when the adenosyl moiety of one ATP (adenosine triphosphate) molecule is transferred to methionine by the action of MAT (ATP-l-methionine *S*-adenosyltransferase). AdoMet is a highly energetic compound as a result of a sulfonium bond between the 5′-carbon atom of the ribose and the sulfur atom of the amino acid [10]. AdoMet is the methyl-donor for the majority of cellular methylation reactions, which are catalyzed by specific methyltransferases targeting important biomolecules, such as DNA, RNA, proteins, and lipids [11]. Interestingly, DNA and protein (e.g., histone) methylations are important epigenetic features that play a critical role in gene expression and regulation [12], and epigenetic dysregulation is implicated in several pathologies. Nevertheless, in mammals, most methyl groups transferred from AdoMet are used in creatine formation, phosphatidylcholine synthesis, and the generation of sarcosine from glycine [8]. Following the transfer of a methyl group to an acceptor molecule, AdoMet is converted into AdoHcy. The latter is further converted into Hcy and adenosine by AdoHcy hydrolase, which is widely distributed in mammalian tissues. The formation of Hcy from methionine is the only pathway for Hcy biosynthesis in humans [13]. Interestingly, this reaction is reversible, and AdoHcy synthesis is strongly favored over its hydrolysis; however, both Hcy and adenosine are rapidly removed under physiological conditions, thus favoring the hydrolysis reaction. Nevertheless, if Hcy accumulates, AdoHcy will accumulate as well, potentially inhibiting cell transmethylation reactions [12].

Hcy can be metabolized via two alternative pathways: it may be irreversibly degraded through the transsulfuration pathway, or remethylated back to methionine via the remethylation pathway. Transsulfuration is the main route for methionine disposal, through which the sulfur atom is integrated into the cysteine molecule [14]. Transsulfuration occurs mainly in the liver and kidney, and it begins with the condensation of Hcy with serine to form cystathionine via cystathionine β-synthase (CBS) with pyridoxal phosphate (PLP) acting as a cofactor. Cystathionine is cleaved to cysteine and α-oxobutyrate by another PLP-requiring enzyme, cystathionine gamma-lyase (CSE). In addition to protein synthesis, cysteine is used in the synthesis of glutathione, an important cellular antioxidant [15]. Otherwise, oxidation of the sulfur atom of cysteine into sulfate further occurs through a number of enzymatic reactions, and a major part is excreted as urinary inorganic sulfate [16]. 

Alternatively, Hcy is remethylated to methionine by either 5-methyltetrahydrofolate (5-methylTHF) or betaine as a methyl donor. In the first case, the methylcobalamin-containing methionine synthase (MS) catalyses the methyl transfer from 5-methylTHF to Hcy, forming methionine and tetrahydrofolate (THF). This reaction, which occurs in almost all cells, involves the formation of enzyme-bound methylcobalamin (methylCbl) [17]. At this point, Hcy metabolism is biochemically linked to intracellular folate metabolism. MS converts the circulating form of folate, 5-methylTHF, into THF, which can then support a variety of cellular reactions. Importantly, these include polyglutamation, which is required to retain intracellular folate. THF is further converted to 5,10-methylenetetrahydrofolate (5,10-methyleneTHF) by serine hydroxymethyltransferase (SHMT), a reaction that requires serine and PLP as a cofactor. After reduction by 5,10-methylenetetrahydrofolate reductase (MTHFR), 5,10-methyleneTHF is converted into 5-methylTHF, available for Hcy remethylation. MTHFR uses FAD (flavin adenine dinucleotide) as a cofactor [18]. Due to the fact that the circulating form of folate is 5-methylTHF, and because the reaction catalyzed by MTHFR is not reversible, folates entering the cells must undergo the MS reaction in order to generate THF and other functionally reduced folates, including those needed for both purine and pyrimidine metabolism [19]. Notably, the MS reaction is methylCbl-dependent. Thus, Cbl deficiency may interfere with the intracellular folate cycling and lead to the accumulation of 5-methylTHF and the depletion of other folate derivatives [19]. 

Betaine-dependent remethylation recycles Hcy into methionine by using non-folate methyl donors and the enzyme betaine-homocysteine methyltransferase (BHMT). BHMT is expressed primarily in the liver and kidney, and uses betaine (trimethylglycine) as a methyl donor. 

Several B vitamins are cofactors in Hcy metabolism. MethylCbl, one active form of vitamin B12, is the cofactor for MS; FAD, one form of vitamin B2, is the coenzyme for MTHFR and MS; FMN, another active form of vitamin B2, is the coenzyme for MS; folates (vitamin B9) are co-substrates in the folate-dependent Hcy remethylation folate pathway; and finally, PLP, the active form of vitamin B6, is the coenzyme for CBS, γ-cystathionase, and SHMT.

The intracellular concentration of Hcy is under tight control [16]. As mentioned, AdoHcy accumulation due to increased Hcy levels can potentially disturb vital transmethylation reactions. In fact, owing to the kinetic characteristics of the AdoHcy hydrolase reaction, intracellular Hcy concentrations should be kept within strict limits. The optimal Hcy concentration in cells is maintained or re-established through folate-dependent remethylation. Exceptions are liver and kidney cells, which can also rely on the folate-independent remethylation and transsulfuration pathways. In addition, cells can also export Hcy to maintain its optimal intracellular levels. However, the mechanisms that regulate Hcy export are not completely understood [4]. Nevertheless, a mechanism involving the removal of the reduced Hcy form (with a free thiol group) to the extracellular compartment has been proposed [20]. A separate mechanism appears to be responsible for the import of oxidized, disulfide forms of Hcy, into cells [20]. In plasma, the majority of Hcy is in disulfide form, as reduced Hcy is rapidly oxidized, reacting with free thiol-containing molecules (including small thiol molecules such as Hcy or cysteine, and proteins with free cysteines, such as albumin) [10,20]. Only a minor part of plasma Hcy remains in its reduced form [20,21]. The widely used biochemical parameter to monitor Hcy is total plasma Hcy (tHcy), which includes the sum of all circulating Hcy molecules, either in its reduced or oxidized forms.

## 3. Endothelial Dysfunction and Atherosclerosis in Hyperhomocysteinemia (HHcy)

The endothelium is the main regulator of vascular wall homeostasis, exerting various functions, including the regulation of vascular tone, permeability, coagulation and fibrinolysis, inflammation, and cell growth [23,24]. Impairment of these functions sets in motion a cascade of events that start with the inability of the endothelium to regulate vascular relaxation and/or cell redox balance [25], termed endothelial dysfunction, which can then trigger an inflammatory response that is usually designated as endothelial activation [26,27]. The activation of endothelial cells results in the upregulation of adhesion molecules and chemokines, which mediate the recruitment of circulating monocytes [27]. Upon infiltration into the intima, monocytes are activated into macrophages that internalize modified lipoproteins (foam cells) [26,28]. In addition, the cytokines and growth factors released by the endothelium act in neighboring tissue, leading to a structural remodeling of the atherosclerotic lesion and to the establishment of a fibromuscular plaque that can further progress to the formation of a fibrous cap, overlying a lipid-rich, necrotic core consisting of oxidized lipoproteins, cholesterol, and cellular debris [29]. In a later stage, the endothelial pro-inflammatory state and apoptosis may contribute to the plaque’s structural instability and rupture, with luminal release of the highly thrombogenic contents of the necrotic core, and possibly leading to an atherothrombotic vascular occlusion [30].

Evidence shows that Hcy exerts its adverse effects by disturbing endothelial function [31,32,33,34,35,36,37]. More specifically, Hcy can impair the endothelium’s capacity to regulate vascular tone. Endothelial cells maintain the vascular tone by releasing mediators such as nitric oxide (NO), prostacyclin, endothelin-1 (ET-1), and thromboxane [38]. Mechanisms by which Hcy reduces the bioavailability of the vasodilator NO are discussed in the next section. In addition, Hcy was positively correlated with ET-1, a potent vasoconstrictor, in subjects with disturbed glucose metabolism and Behçet’s disease [39]. Enhanced thromboxane biosynthesis was also observed in patients with high plasma Hcy [40]. Besides being a vasoconstrictor, thromboxane induces platelet production [41]. Platelets can also play a role in endothelial dysfunction (via chemokine release) and contribute to the rupture of the vulnerable plaque (via angiogenesis activation and thrombus formation) [41]. A meta-analysis study of human ischaemic heart disease reported that seventeen studies (out of twenty-two) showed a statistically significant effect of Hcy on increasing pro-thrombotic platelet function (increased platelet activation), thromboxane production, and platelet aggregation [42].

A shift towards decreased NO and increased reactive oxygen species (ROS) dictates the trigger for endothelial dysfunction and atherogenesis. Reduction in NO production can contribute to endothelial activation through the upregulation of adhesion molecules, such as intracellular adhesion molecule-1 (ICAM-1), vascular adhesion molecule-1 (VCAM-1), E-selectin, and macrophage chemoattractant peptide-1 (MCP-1) [43]. Likewise, an increase in oxidative stress can stimulate the expression of cytokines and adhesion molecules in endothelial cells via the NF-κB pathway [33]. Sustaining a snowball-like effect, oxidative stress can further lead to the oxidative inactivation of NO and eNOS (endothelial NO synthase) uncoupling, further decreasing NO bioavailability [44,45] and contributing to the formation of more ROS [46,47].

Either directly or via ROS production, Hcy has been associated with vascular inflammation [5,33], another main feature of endothelial dysfunction. Inflammatory markers are used to monitor atherosclerotic disease progression, and treatments for patients with atherosclerosis operate, at least partly, via modulation of immune responses [23,25]. Cell culture studies have shown that Hcy induces the production of pro-inflammatory cytokines, such as MCP-1 and IL-8, through the activation of NF-κB [48]. NF-κB is a transcription factor that stimulates the production of cytokines, chemokines, and adhesion molecules, thus contributing to endothelial activation, leukocyte recruitment, and promoting atherogenesis [49]. Moreover, we have shown that, in endothelial cells, the precursor of Hcy; AdoHcy, activates NF-κB and induces the expression of key pro-inflammatory molecules, including IL-1β, ICAM-1, VCAM-1, and E-Selectin [33]. Studies using hyperhomocysteinemic apolipoprotein E (apoE) -deficient mice (an animal model of atherosclerosis) also revealed the activation of NF-κB and downstream proinflammatory mediators in atherosclerotic lesions, supporting the role of inflammation in HHcy-associated atherogenesis [50,51].

Endothelial cell apoptosis is a hallmark of atherosclerotic lesions and contributes to the formation and rupture of atherosclerotic plaque. Hcy can also contribute to atherogenesis by mediating apoptotic cell death of endothelial cells and smooth muscle cells, as shown in studies using human cultured cells [32,49,52]. Moreover, circulating apoptotic endothelial cells were detected in hyperhomocysteinemic patients [5]. Additional studies using human endothelial cells show that increased ROS, endoplasmic reticulum stress, and Hcy-thiolactone formation contribute to the observed Hcy-induced apoptosis [5]. 

## 4. Mechanisms Induced by HHcy Associated with Endothelial Dysfunction and Atherogenesis

**Figure 2:**
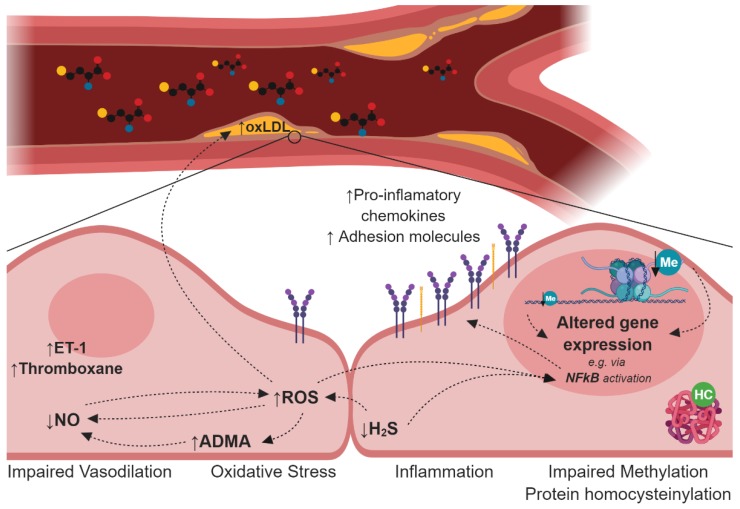
Schematic representation of the leading mechanisms proposed to underlie the implication of Hcy elevation in endothelial dysfunction and CVD: (i) impaired vasodilation, due to decreased NO bioavailability and increased vasoconstrictor molecules, as ET-1 and thromboxane; (ii) oxidative stress, either due to impaired antioxidant systems, uncoupled eNOS, and/or increased H_2_S. Hcy-induced ROS formation can contribute to LDL oxidation as well as to the activation of the endothelium; (iii) via upregulation of adhesion molecules and pro-inflammatory cytokines. Both ROS and H_2_S are known activators of the NF-κB complex, which induces the expression of various pro-inflammatory genes; and (iv) the impaired methylation reactions and increased *N*-homocysteinylation can further contribute to impaired protein expression/stability and/or activity. This figure was created using BioRender. ADMA: asymmetric dimethylarginine; ET-1: endothelin-1; HC: *N*-homocysteinylation; Me: methylation; NO: nitric oxide; oxLDL: oxidized low-density lipoprotein; ROS: reactive oxygen species.

The long-standing association between HHcy, endothelial dysfunction, and CVD has stimulated intense research efforts, resulting in the development of several hypothesis, including the interference of Hcy in nitric oxide production, deregulation of the hydrogen sulfide signaling pathway, oxidative stress, disturbances in lipoprotein metabolism, protein *N*-homocysteinylation, and cellular hypomethylation (Figure 2).

### 4.1. Impairment of the Nitric Oxide Synthesis

NO, the key vasodilator factor in endothelium, is produced by oxidation of arginine through the catalytic activity of nitric oxide synthase (NOS). This reaction requires NADPH and O_2_ as co-substrates and yields NO and citrulline as end products. Importantly, NOS is inhibited by methylated analogues of arginine, namely *N*-monomethylarginine (l-NMMA) and asymmetric dimethylarginine (ADMA) [53], which are synthesized in vivo by the action of a family of enzymes known as protein arginine methyltransferases. Proteolysis of proteins containing l-NMMA and ADMA release them into the cytosol from where they pass out of the cell into blood. Agreeably, elevation of circulating ADMA has been extensively associated with atherosclerotic vascular disease [54], and also multiple organ failure syndromes [55], pulmonary hypertension [56], stroke [57], sickle cell disease [58], among other pathologies. The apparent lack of atherogenicity of l-NMMA may be explained by the fact that its concentration in plasma is typically ten times lower than that of ADMA [59]. Another guanidino-substituted analogue of arginine, symmetric dimethylarginine (SDMA), does not inhibit NOS activity [53], but it competes with arginine for its cationic amino acid transporter, and thus its elevation can potentially compromise intracellular arginine availability and NO production [60]. Free l-NMMA, ADMA, and SDMA may be cleared from the body by renal excretion, but most l-NMMA and ADMA are hydrolyzed to dimethylamine and citrulline via dimethylarginine dimethylaminohydrolase (DDAH) activity [61]. Oxidative stress has been shown to reduce DDAH activity, contributing to elevation of ADMA [62]. By contrast, in a mouse model, global overexpression of DDAH1, the predominant DDAH isoform in kidney and liver, reduces ADMA levels and increases NO production [63]. Consistent with an essential role of DDAH activity in maintaining vascular homeostasis, these mice manifest enhanced endothelial repair after vascular injury [64].

Since mild elevation of both tHcy and ADMA has been associated with incidence of cardiovascular disease, it is tempting to speculate that the detrimental effect of elevated tHcy on the endothelium may be mediated by ADMA. Interestingly, monkeys fed a methionine-rich diet displayed elevation in both amino acids and reduced NO-dependent carotid artery vasodilatation [65]. Furthermore, an increase in methionine concentration in the medium of an endothelial cell culture model resulted in increased formation of both ADMA and tHcy [66]. A possible mechanism associating Hcy and ADMA was proposed in a study where the treatment of endothelial cells with Hcy significantly suppressed DDAH and NOS activity, and reduced NO production, which was attributed to decreased expression of DDAH2, the major DDAH isoform in endothelial cells [67]. Therefore, suppression of DDAH2 expression may contribute to Hcy-induced endothelial dysfunction. It is also possible that oxidative stress induced by Hcy enhances the degradation of DDAH2. Further studies are needed to understand whether elevation of tHcy does indeed parallel that of ADMA in the context of endothelial dysfunction and, if so, to uncover the mechanisms responsible for this association. 

### 4.2. Deregulation of the Hydrogen Sulfide Signalling Pathway

Dihydrogen sulfide (or sulfane), commonly known as hydrogen sulfide (H_2_S), has long been known as a toxic gas or environmental pollutant with a rotten egg odor [68]. In recent years, it has become evident that H_2_S is produced endogenously and that it is a signaling molecule that regulates several physiological processes, including in the vascular system where it participates in the fine regulation of endothelial homeostasis [69,70,71,72,73,74]. Two enzymes of the transsulfuration pathway, CBS and CSE, produce H_2_S [75,76]. As mentioned earlier, the canonical role of these enzymes is to convert methionine to cysteine via the transsulfuration pathway. However, CBS and CSE display significant substrate ambiguity and relaxed reaction specificity resulting in a multitude of possible reactions, many of which generate H_2_S [68]. Both CBS and CSE are the major source for vascular H_2_S production [69,70,71,72,73,74]. More specifically, CBS catalyzes H_2_S synthesis from cysteine and Hcy, and CSE catalyzes the production of pyruvate and thiocysteine [77]. Thiocysteine is then non-enzymatically decomposed to H_2_S and cysteine [78]. The expression of the two major enzymes involved in H_2_S biogenesis is tissue-specific. Under normal conditions, CBS is the dominant H_2_S-producing enzyme in the cerebrovascular system, and the CSE/H_2_S pathway has a crucial role in the cardiovascular system [79]. Very recently, an additional link between Hcy metabolism and H_2_S was proposed when sulfane sulfur was implicated in the regulation of MS activity, modulating the B12 and folate-dependent remethylation of Hcy to methionine [80].

H_2_S exerts its beneficial biological effects on the endothelium acting on different signaling pathways via persulfidation, an oxidative posttranslational modification of cysteine residues (RSH) to persulfides (RSSH) [68]. For instance, H_2_S inhibits vascular inflammation [75,81] by inhibiting the NF-kB pathway and by activating potassium and calcium channels [76,82]. Moreover, H_2_S decreases ROS levels in endothelial cells by scavenging ROS and upgrading antioxidant defense machinery [83,84,85,86,87]. In fact, the expression of many antioxidant enzymes, such as catalase, superoxide dismutase, glutathione peroxidase, and glutathione-S-transferase, is upregulated by H_2_S [88]. 

Lastly, and similar to the overall function of NO in the endothelium, H_2_S causes vasorelaxation [89,90], participates in the physiological maintenance of blood pressure [91], and serves an endogenous stimulator of angiogenesis [92,93]. Over the last years, several lines of data have indicated that these two pathways, NO and H_2_S, cooperate with each other to maintain homeostasis in the vascular system [94]. The vasodilatory effect of H_2_S and NO observed on rat aortas, was one of the first studies suggesting the existence of a functional crosstalk between the two gasotransmitters [95]. Since then, there has been growing evidence that the vasodilatory effects of H_2_S are intricately linked to NO signaling pathways [94].

Consistent with its important role in endothelial homeostasis, anti-atherosclerotic properties of H_2_S have been reported. Furthermore, H_2_S deficiency appears to accelerate atherosclerosis. CSE is one of the key enzymes producing endogenous H_2_S and is expressed abundantly in the mammalian cardiovascular system. When fed an atherogenic diet, CSE knockout mice were shown to have lower aortic H_2_S production and to develop a higher number of aortic vascular lesions than wild-type mice [91]. In another example, expression of the adhesion molecule ICAM-1 was significantly elevated in the aorta of CSE knockout mice on an atherogenic diet [96]. On the contrary, supplementation with H_2_S inhibits atherosclerosis. For instance, H_2_S exerted an anti-atherogenic effect and inhibited ICAM-1 expression in apoE knockout mice. In the same study, H_2_S inhibited ICAM-1 expression in TNF-alpha-induced human umbilical vein endothelial cells (HUVECs) via the NF-κB pathway [97]. In another example, exogenous H_2_S decreased vascular inflammation, oxidative stress, and atherosclerotic plaque formation in apoE knockout mice [98]. Lastly, increased endogenous H_2_S production via *CSE* activation was associated with reduced atherosclerosis in the same animal model [99].

Interestingly, disturbed H_2_S bioavailability has been suggested to reveal the progress and prognosis of endothelial dysfunction associated with HHcy [100]. In fact, several pieces of evidence have shown that HHcy causes downregulation of CSE and CBS, resulting in H_2_S depletion [100]. Decreased H_2_S disarms the endothelium from H_2_S protection, which in turn leads to deterioration of endothelial function and subsequently to the vascular disease associated with HHcy [100]. CBS deficiency decreased H_2_S production in cultured endothelial cells [101]. Moreover, exogenous H_2_S corrected endothelial dysfunction in vivo [102], and was able to protect these cells from HHcy-induced damage [103]. Taken together, these findings suggest that CBS deficiency will contribute to endothelial dysfunction by decreasing H_2_S-induced vascular relaxation [101,104]. However, in the liver, absence of CBS can paradoxically augment H_2_S production by CSE [105]; in the heart, negative feedback regulation of CBS and CSE was reported [79], where HHcy suppresses CBS, thereby upregulating CSE and increasing H_2_S production. Overall, these observations suggest that the in vivo effects of CBS deficiency on vascular H_2_S and endothelial function warrant further investigation [4].

### 4.3. Oxidative Stress

A large body of evidence emphasizes the significant role of oxidative stress in Hcy-induced endothelial dysfunction and atherosclerosis. Oxidative stress is commonly defined as an imbalance between the formation of reactive species and the antioxidant capacity of the cell [106]. Findings in patients and animal models show that HHcy can induce oxidative stress via different molecular mechanisms (Table 1), either by modulating reactive oxygen species (ROS) production or by impairing relevant antioxidant systems [107].

The most physiologically relevant ROS include the superoxide anion (O^2−^.), the hydroxyl radical (●OH), and hydrogen peroxide (H_2_O_2_). The autoxidation of Hcy can generate ●OH and H_2_O_2_ [108]. However, the role of autoxidation in Hcy-induced cell oxidative stress has been questioned, due to the fact that other thiols—such as cysteine—are present at much higher concentrations than Hcy, which can also undergo autoxidation and are not associated with impaired redox balance [109]. Cellular superoxide anions are mainly generated by nicotine adenine dinucleotide phosphate (NADPH) oxidase, xanthine oxidase, or by the mitochondrial electron transport system [106]. Hcy was shown to upregulate the expression of NADPH oxidase in HHcy mice, thus contributing to an increase in superoxide anion production [110]. NADPH oxidase is codified by NOX (NADPH oxidase) family genes. NOX2 is the predominant isoform in the endothelium, and a correlation between Hcy levels and NOX2 expression has been found in human endothelial cells and rat cardiomyocytes [52,111]. NADPH oxidase likely accounts for just a part of the ROS produced during HHcy [5]. Furthermore, in cell culture and animal models, Hcy has been linked to endothelial nitric oxide synthase (eNOS) uncoupling, which results in the production of superoxide anion rather than nitric oxide (NO) [112,113]. The release of ROS, as superoxide, can activate other ROS producers and further contribute to oxidative stress [5]. Superoxide anions can be converted into H_2_O_2_ via superoxide dismutase (SOD), and can react with NO to generate peroxynitrite (ONOO^−^). Superoxide and H_2_O_2_ are known activators of NF-κB and can potentiate the pro-inflammatory response associated with HHcy. In cultured endothelial cells, Hcy has also been associated with increased H_2_O_2_, either directly [114,115] or via *S*-adenosylhomocysteine accumulation [34]. 

The hydroxyl radical can be generated through the breakdown of H_2_O_2_ [116]. It is the most reactive of the cellular ROS and can lead to the oxidation and damage of proteins, lipids, carbohydrates, and DNA [106]. Due to its high reactivity, the hydroxyl radical contributes to the oxidation of low-density lipoproteins (LDL), which is critical in the atherosclerotic process. An association between Hcy-induced hydroxyl radical generation and oxidation of lipids in LDL was found in endothelial cells [117,118]. 

Several studies also highlight the relevance of antioxidant systems loss in Hcy-mediated oxidative stress [34,107,119,120,121]. Cellular antioxidant systems are commonly defined as enzymatic and non-enzymatic antioxidants. The first comprises enzymes that neutralize ROS and decrease oxidative stress, such as SODs, catalase, glutathione peroxidase (GPx), thioredoxin, and peroxiredoxin; the non-enzymatic antioxidants include ROS scavengers, such as glutathione, and vitamins A, C, and E [106]. 

High Hcy levels have been previously associated with decreased glutathione [122], a key cellular antioxidant, in cardiac patients, as well as with a reduction in vitamins B12 and E in HHcy patients [107]. GPx (GPx-1 and GPx-2), SOD (SOD1 and SOD2), and thioredoxin expression were also found reduced in hyperhomocysteinemic mice and endothelial cells [107,108,120,123]. Moreover, a recent study including hypertensive hyperhomocysteinemic patients showed that patients with high tHcy levels had lower plasma SOD and catalase when compared to patients with normal plasma Hcy [119]. 

The molecular mechanisms behind Hcy-associated changes in the expression of ROS producers or antioxidant proteins are mostly unclear. Our group and others have highlighted the importance of altered methylation potential mediated by AdoHcy during HHcy. The methylation imbalance that is mediated by AdoHcy accumulation may, for example, disturb epigenetic mechanisms that regulate the expression of key players on cell redox balance [4,33,34,124,125]. Using human endothelial cells, we have shown that AdoHcy-mediated hypomethylation contributes to GPX-1 downregulation, a central antioxidant system in the endothelium [34].

### 4.4. Disturbances in Lipoprotein Metabolism

Atherosclerosis is a highly multifactorial disease, and disturbances in lipid and lipoprotein metabolism are linked to a high risk of disease [126]. Lipoproteins are mainly responsible for the transport of lipids—cholesterol and triacylglycerols—in blood. Low-density lipoproteins (LDL) and high-density lipoproteins (HDLs) are the most abundant lipoproteins in plasma, and they have been a critical target for the development of therapies against cardiovascular diseases [127]. Upon endothelial dysfunction, atherogenic lipoproteins, as LDLs, enter the intima, where they undergo oxidation (oxLDL) and aggregate within the extracellular intimal space, thereby increasing their uptake by macrophages and leading to the formation of foam cells, a central component of the atherosclerotic plaque [127]. As previously mentioned, one of the most reliable links between HHcy and vascular disease is oxidative stress. ROS generated in HHcy can contribute to the oxidation of LDL and promote its accumulation in the atherosclerotic lesion. Different reports have shown that Hcy causes oxidative stress and leads to oxidation of LDL [117,128]. 

LDL oxidation facilitates its uptake by foam cells via the scavenger receptor family. Griffiths and co-workers showed that Hcy released from methionine-loaded HUVECs promotes LDL protein nitration [117]. Hcy-induced LDL nitration is also associated with enhanced monocyte uptake [117]. Similar to oxidation and acetylation, other LDL post-translational modifications are associated with an increased uptake by monocytes/macrophages. LDL modifications include oxidation, acetylation, glycation, methylation, etc. High Hcy levels were also associated with increased uptake of acetylated-LDL in mice [129]. In contrast to other modifications, methylated-LDL abolishes its recognition by LDL receptors [130]. HHcy-associated hypomethylation, due to increased AdoHcy, may contribute to reduced LDL methylation and increased uptake by monocytes or activated macrophages in the atherosclerotic lesion [129]. 

As for LDL, cholesterol is the primary lipid constituent of HDL. However, in contrast to LDL, plasma concentrations of HDL are inversely associated with atherosclerotic disease [127]. HDL is the main lipoprotein involved in the removal of excess cholesterol from peripheral tissues, delivering it to the liver to be redistributed to other tissues or removed from the body (reverse cholesterol transport) [131]. Besides promoting modified LDL uptake and, thus favoring atherogenesis, Hcy is associated with impaired HDL metabolism. Mice with HHcy presented decreased HDL-cholesterol levels, as a consequence of a reduction of HDL-cholesterol production rate [132,133]. Plasma tHcy concentrations were also found to negatively correlate with HDL-cholesterol in patients with coronary artery disease [134]. HDL reduction in HHcy suggests an impairment in the reverse cholesterol transport, which can constitute an additional mechanism by which Hcy is linked to atherosclerosis.

### 4.5. Protein N-Homocysteinylation

During protein biosynthesis, Hcy can be mistakenly used by methionyl-tRNA synthetase to generate homocysteine thiolactone (HTL), a cyclic thioester that rapidly reacts with proteins by forming amide bonds with amino groups of lysine residues [135]. The ensuing generation of *N*-homocysteinylated proteins with altered structure and biochemical properties has been suggested to contribute to the vascular pathology associated with HHcy [136]. In fact, a study conducted in HUVECs showed that Hcy supplemented to the medium was converted into HTL, and the extent of this conversion was proportional to the concentration of Hcy. In addition, supplementation of folic acid inhibits HTL biosynthesis by lowering tHcy [137].

The cytotoxicity of HTL was clearly demonstrated by the observation that its supplementation to endothelial culture medium induces apoptosis in a concentration-dependent manner [138]. In a gene expression profiling study, gene set enrichment analysis identified chromatin organization, one-carbon metabolism, lipid-related processes, and blood coagulation among the top molecular pathways significantly affected by HTL in HUVECs, and differentially expressed genes were also enriched in the ‘atherosclerosis, coronary heart disease’ disease ontology [139]. Other recent in vitro studies showed that HTL inhibits insulin receptor tyrosine kinase activity, thereby inhibiting the phosphorylation of phosphatidylinositol 3-kinase and glycogen synthesis [140,141]. This effect could be the basis for the association between HHcy, hyperinsulinemia, and CVD [142,143]. In addition, fibrinogen—a key blood coagulation protein—is modified by HTL, thus trigger pro-thrombotic changes in fibrin clot structure and stability [144,145]. 

HTL is elevated in patients with HHcy due to pathogenic mutations in either *MTHFR* or *CBS* [146]. In addition, enhanced protein *N*-homocysteinylation was shown in patients with recurrent venous thromboembolism and to possibly underlie the enhanced inflammatory state associated with this condition [147]. The level of urinary HTL has been recently proposed as a novel acute myocardial infarction risk predictor, independent of the presence of traditional risk factors [148]. Moreover, plasma HTL levels have been associated with risk of coronary heart disease [149]. Interestingly, in the same study, a frequent polymorphism in *PON2*, encoding a cellular antioxidant enzyme of the paraoxonase family that protects cells against oxidative stress, was associated with accumulation of HTL [149]. Similarly, a frequent polymorphism in *PON1*, which encodes a cardio-protective enzyme known to hydrolyze HTL in vitro, was shown to elicit elevation of urinary HTL levels [150]. Homocysteine thiolactonase activity was also negatively associated with the thickness of the carotid intima media in patients with type 2 diabetes mellitus [151]. Therefore, metabolic conversion of Hcy to HTL and protein N-homocysteinylation by HTL likely plays a role in Hcy-induced vascular damage.

### 4.6. Cellular Hypomethylation

Hcy metabolism plays a determinant role in the regulation of cellular methylation capacity. As mentioned earlier, a noteworthy aspect to bear in consideration is that the hydrolysis of *S*-AdoHcy to Hcy and adenosine is reversible, and the synthesis of AdoHcy is thermodynamically favored. Under normal physiological conditions, the reaction proceeds in the hydrolysis direction due to rapid clearance of Hcy and adenosine by metabolic conversion and cellular export [9]. However, defects in the transsulfuration and remethylation pathways leading to HHcy cause elevated AdoHcy, as substantiated by several observations in humans, animal models, and cell culture studies [124,152,153,154]. Since AdoHcy is a potent endogenous inhibitor of cellular methyltransferases, the setting of HHcy favors a hypomethylating environment, compromising important methyl transfer reactions involved in vascular homeostasis. 

The detrimental impact of Hcy and AdoHcy elevation on DNA methylation status has been extensively studied (Table 2). For instance, moderate elevation of plasma tHcy levels in humans is associated with elevated AdoHcy levels and decreased DNA methylation in lymphocytes/leukocytes [152,153]. These observations in humans are mirrored by observations in animal models, for example global DNA hypomethylation was found in atherosclerotic lesions in rabbits and mice [155,156]. DNA methylation is a crucial mechanism for epigenetic regulation of gene expression. Therefore, AdoHcy-determined low cellular methylation capacity can lead to impaired DNA methylation and contribute to the disturbed gene expression patterns associated with endothelial dysfunction in the context of HHcy. Several studies focusing on specific loci support this hypothesis. Hcy has been shown to induce enhancer CpG hypomethylation upstream of the typically imprinted *H19* gene in human vascular smooth muscle cells cultured in vitro [157]. In addition, in patients with renal disease, HHcy led to a shift from monoallelic to biallelic expression of *H19* [158], and in CBS-deficient mice, the expression of *H19* was also significantly increased [159]. Other loci with cis-regulatory elements whose methylation state has been shown to be affected by Hcy and/or AdoHcy elevation include the pro-angiogenic factor *PDGF* (platelet-derived growth factor) [160], genes involved in cell cycle progression (e.g., *cyclin A* [161] and *BNIP3* [162]), genes involved in cholesterol metabolism (e.g., *SREBF1* and the LDL receptor gene) [163], vascular inflammatory response genes such as *IL1B*, *IL6*, *IL8*, and *ICAM1* [33,164], the gene encoding the extracellular antioxidant SOD [155], a primary extracellular scavenger of superoxide in the blood vessel wall, and the promoter of the gene encoding the human telomerase reverse transcriptase (hTERT) [165]. The contribution of DNA methylation disturbance to the vascular pathology associated with Hcy elevation has been the subject of vigorous research efforts, and it is beyond the scope of this paper to discuss it in detail. For a more thorough discussion of this exciting topic, we encourage the reader to refer to the recently published comprehensive reviews [4,166].

Although DNA methylation has merited most of the attention, the impact of Hcy metabolism disturbance on other methylation reactions may be of equal importance (Table 2). One such methyl transfer reaction is that of RNA, a burgeoning topic in basic and biomedical research. Most knowledge of the molecular function of RNA methylation has been shaped by work on abundant RNA species such as transfer RNAs (tRNAs) and ribosomal RNAs (rRNAs) [167,168], but recent technological breakthroughs, including next-generation sequencing (NGS) and mass spectrometry applications, have extended research on this topic to include other RNA species and have enabled the identification of several methylated nucleotide derivatives. Interestingly, adenosine RNA methylation marks can either be removed enzymatically [169,170] or subjected to further modifications [171], showing that RNA methylation is a dynamic process that might be involved in cellular adaptive mechanisms. Another player in this dynamic regulation is metabolism, including Hcy metabolism. Similar to DNA methyltransferases, RNA methyltransferases use AdoMet as methyl donor compound and are inhibited by AdoHcy [172]. Nevertheless, little attention has been dedicated to the possibility that RNA methylation is disturbed in HHcy. Our findings have shown that AdoHcy accumulation in cultured human endothelial cells decreases the methylation extent of the selenocysteine-carrying tRNA (Sec-tRNA), leading to altered expression of a subset of selenoproteins [34]. These proteins include GPx-1, a critical selenoprotein involved in cell detoxification and redox regulation that was previously linked to endothelial dysfunction in the context of HHcy [173]. 

Disturbance of Hcy metabolism also affects protein methylation (Table 2), a posttranslational modification that participates in vital processes as transcription control, RNA processing, DNA repair, telomere maintenance, and signal transduction [174,175]. Notably, intracellular accumulation of AdoHcy in HUVECs elicits protein arginine hypomethylation to a more significant extent than DNA hypomethylation [176]. In our hands, global protein arginine methylation was found to be decreased in tissues of two murine models of HHcy concomitant with a decreased AdoHcy/AdoMet ratio [124,125]. In an early study, low protein carboxyl methylation of the erythrocyte membrane proteome in chronic renal failure patients was attributed to AdoHcy elevation [177]. In the same study, a specific protein involved in membrane stability and integrity, ankyrin, was identified as being especially sensitive to the loss of cellular methylation capacity [177]. A subsequent study using vascular endothelial cells reported the Hcy-induced inhibition of carboxyl methylation of p21(ras) in association with growth inhibition [178]. Arginine methylation of the peroxisome proliferator-activated receptor gamma coactivator 1-alpha (PGC-1α), a transcriptional coactivator that regulates genes involved in energy metabolism, was also found to be impaired in the myocardium of weaning rats whose dams were subjected to dietary methyl donor deficiency [179]. Histone methylation, an essential chromatin modification that is also involved in epigenetic gene regulation, is also affected by disturbed Hcy metabolism. In a recent animal study, the status of methionine availability in the liver was correlated with the levels of the classical activation mark H3K4me3 (trimethylation of lysine 4 on histone H3), with matching changes in gene expression [180]. The repressive marks H3K9me2 (dimethylation of lysine 9 on histone H3), H3K27me2 (dimethylation of lysine 27 on histone H3), and H3K27me3 (trimethylation of lysine 27 on histone H3) were decreased in advanced atherosclerotic plaques in human vessels [181,182]. In endothelial cell studies, we have shown that AdoHcy accumulation decreases global H3K27me3 content and activates the pro-inflammatory NF-κB pathway, leading to increased expression of adhesion molecules and inflammatory cytokines reminiscent of a pro-atherogenic environment [33]. These observations show that a disturbed cellular methylation capacity in the setting of HHcy results in protein hypomethylation, which may contribute to endothelial dysfunction.

The importance of the impairment of cellular methylation reactions in the setting of HHcy due to the accumulation of AdoHcy merits further consideration, and can be extended to substrates other than those at the DNA–RNA–protein axis, such as lipids, hormones, and neurotransmitters. 

## 5. Controversy Regarding the Negative Results from Hcy-Lowering Human Trials

In recent years, the long-standing notion that Hcy elevation in blood has a causal role in establishing vascular disease phenotypes has been challenged by the disappointing results of B-vitamin dietary interventions, which lowered Hcy levels but did not yield the anticipated cardioprotective effects. In fact, randomized trials among patients with pre-existing CVD have failed to support the benefits of such Hcy-lowering therapy on disease risk [183,184,185]. It has been proposed that the Hcy-lowering effects of B vitamins are offset by its deleterious effects, including the proinflammatory and proliferative effects on advanced atherosclerotic lesions [186]. While vitamin B supplementation effectively lowers circulating Hcy levels, it is not known whether such effect mirrors a decrease in intracellular Hcy levels. In the latter case, the cytotoxic effects of Hcy on the endothelium, such as disruption of intracellular redox status, protein N-homocysteinylation, and global cellular hypomethylation, would remain operative. It is also possible that Hcy exerts an initial detrimental effect on the vascular wall and that this effect persists via epigenetic maintenance mechanisms that are not suppressed by Hcy-lowering therapy.

## 6. Conclusions

Epigenetics is a fast-paced area of biomedical research and is unveiling the mechanisms by which genes are regulated in development and disease, particularly in the context of the interplay between metabolism and epigenetics. For instance, Hcy metabolism and histone methylation are biochemically linked, since histone methyltransferases require AdoMet as a methyl group donor. As discussed earlier, AdoHcy is the Hcy metabolic precursor and a negative regulator of AdoMet-dependent methyltransferases, (including histone methyltransferases) [187]. More importantly, AdoHcy has been claimed to be a better indicator of CVD than Hcy [188,189,190]. Furthermore, recent exciting observations highlight the status of Hcy metabolism as a crucial factor determining chromatin dynamics and epigenetics [180,191], and as deep-sequencing approaches become increasingly feasible and cost-effective, the study of this relationship will likely develop and expand to the context of endothelial dysfunction. In summary, the relationship between Hcy metabolism and endothelial dysfunction remains an important and exciting field of research.

## Figures and Tables

**Table 1:** Mechanisms by which HHcy can contribute to oxidative stress.

Molecular Mechanism		Species	References
NADPH oxidase upregulation		*M. Musculus*, *R. norvegicus* (cardiomyoblasts), *H. Sapiens* (cultured ECs)	[52,110,111]
eNOS uncloupling		*R. norvegicus*, *M. Musculus*, *H. Sapiens*	[112,113]
Decreased non-enzymatic antioxidants:	glutathione vitamin B12 vitamin E	*H. Sapiens*	[107,122]
Impaired enzymatic antioxidants:	GPx-1 and -2 Thioredoxin SOD Catalase	*M. Musculus*, *H. Sapiens*	[107,108,119,120,123]

**Table 2:** Summary of observations in peer-reviewed articles linking Hcy metabolism disturbance with impaired cellular methylation.

Species	Observations	References
*H. sapiens*	Correlation between circulating AdoHcy, tHcy and global DNA methylation levels	[152,153]
*H. sapiens*	Hcy-induced enhancer CpG hypomethylation at the imprinted *H19* locus in human vascular smooth muscle cells; biallelic expression of *H19* in patients with renal disease and HHcy	[157,158]
*H. sapiens*	Hcy-induced promoter hypomethylation and mRNA upregulation of the pro-angiogenic gene *PDGF* (platelet-derived growth factor)	[160]
*H. sapiens*	Hcy-induced DNA hypomethylation of cell cycle progression genes *cyclin A* and *BNIP3*	[161,162]
*H. sapiens*	Hypomethylation of *SREBF1* and the LDL receptor gene upon deficiency of vitamin B12 insufficiency	[163]
*H. sapiens*	AdoHcy-induced impaired expression of adhesion molecules and cytokines via inhibition of the EZH2 methyltransferase, leading to decrease in the levels of the repressive histone modification mark H3K27me3	[33]
*H. sapiens*	Hcy-induced accelerated senescence of endothelial cells via hypomethylation of the telomerase reverse transcriptase gene	[165]
*H. sapiens*	AdoHcy-induced hypomethylation of the selenocysteine-carrying tRNA and altered expression of selenoproteins, including the critical redox regulator GPx-1	[34]
*H. sapiens*	AdoHcy-induced global protein arginine methylation	[176]
*H. sapiens*	Loss of protein carboxyl methylation in erythrocytes of chronic renal failure patients due to AdoHcy elevation	[177]
*H. sapiens*	Hcy-induced inhibition of carboxyl methylation of p21(ras) in vascular endothelial cells leading to growth inhibition	[178]
*H. sapiens*	Decrease in the levels of the repressive marks H3K9me2, H3K27me2 and H3K27me3 in advanced atherosclerotic plaques	[181,182]
*R. norvegicus*	Decreased global protein arginine methylation in diet-induced HHcy	[124]
*R. norvegicus*	Loss of protein arginine methylation of the PGC-1α transcriptional coactivator in the myocardium upon methyl donor dietary deficiency	[179]
*M. musculus*	Increased expression of *H19* in CBS-deficient mice	[159]
*M. musculus*	Decrease global protein arginine methylation in HHcy induced by CBS deficiency	[125]
*M. musculus*	Correlation between the levels of the histone modification mark H3K4me3 in liver and methionine availability in diet	[180]
*M. musculus*, *O. cuniculus*	Global DNA hypomethylation in atherosclerotic lesions	[155,156]
*O. cuniculus*	Hypomethylation of the antioxidant extracellular SOD gene in atherosclerotic lesions	[155]
